# “A *calf cannot fail to pick a colour from its mother*”: intergenerational transmission of trauma and its effect on reconciliation among post-genocide Rwandan youth

**DOI:** 10.1186/s40359-023-01129-y

**Published:** 2023-04-07

**Authors:** Marie Grace Kagoyire, Jeannette Kangabe, Marie Chantal Ingabire

**Affiliations:** 1 University of Stellenbosch, the Centre for the study of the afterlife of violence, and the reparative Quest (AVReQ), Stellenbosch, South Africa; 2 Prison Fellowship Rwanda, Kigali, Rwanda; 3 Senior Researcher Community Based Sociotherapy Rwanda, Kigali, Rwanda

**Keywords:** Intergenerational transmission of trauma, Reconciliation, Genocide, Youth, Rwanda

## Abstract

**Background:**

More than one million Rwandans were killed over a span of one hundred days during the 1994 genocide against the Tutsis. Many adult survivors were severely traumatized by the events, and young people, including those who were born after the genocide, have experienced similar genocide-related trauma. Building on a growing body of research on the generational transmission of trauma, our study addressed the following questions: (1) what are the possible mechanisms of trauma transmission from older generation to post-genocide Rwandan youth, and (2) what are the effects of intergenerational trauma on reconciliation processes in Rwanda.

**Methods:**

A qualitative study was conducted in Rwanda among youth born after the genocide, with parents who survived the 1994 genocide against the Tutsis and among mental health and peace-building professionals. Individual interviews (IDIs) included 19 post-genocide descendants of survivors and six focus group discussions (FGDs) were conducted with 36 genocide survivor parents residing in Rwanda’s Eastern Province. Ten IDIs were also conducted with mental health and peace-building professionals in the capital city of Kigali. Respondents were recruited through five local organisations that work closely with survivors and their descendants. An inductive thematic analysis approach was used to analyse the data.

**Results:**

Findings from this study suggest that the trauma experienced by genocide survivor parents is perceived by Rwandan youth, mental health and peace-building professionals, and survivor parents themselves to be transmitted from parent to child through human biology mechanisms, social patterns of silence and disclosure of genocide experiences, and children’s and youth’s everyday contact with a traumatized parent. Genocide-related trauma among survivor parents is seen as often being triggered by both life at home and the annual genocide commemoration events. Additionally, when transmitted to genocide survivor descendants, such trauma is understood to negatively affect their psychological and social well-being. Intergenerational trauma among youth with genocide survivor parents limits their involvement in post-genocide reconciliation processes. Findings specifically show that some youth avoid reconciliation with a perpetrator’s family due to mistrust as well as fear of re-traumatizing their own parents.

## Background

In 1994, Rwanda experienced the genocide against the Tutsis that took nearly one million lives, with most of the victims being Tutsis^1^. The perpetrators of the genocide were mostly from the Hutu ethnic group and were often neighbours, friends, or relatives of the victims [1–3]. The genocide impacted the country in many ways. Millions of people took refuge in neighbouring countries while others were jailed due to being suspected or convicted of genocide crimes. Both victims and perpetrators eventually returned and lived together to rebuild their previously shattered communities [3, 4]. Survivors were severely traumatized. Witnessing dead bodies lying out in the open and in mass graves as well as experiences of fear, mistrust, and isolation following the destruction of social ties and rupture of former sources of support, all characterized Rwandan everyday life after genocide [5]. Furthermore, survivors and perpetrators lived in integrated communities where it was difficult for Rwandans to come to terms with their history [6–8].

### Efforts to reconstruct the country following the 1994 genocide

The Rwanda government implemented many transitional justice initiatives to promote healing and reconciliation between genocide survivors and perpetrators, with the goal of building a peaceful future. For example, mental health services became regarded as an important component of peace-building [9], and many new mental health professionals were trained to respond to the emotional needs of those who directly experienced the genocide [3, 10, 11].

Moreover, the annual genocide commemoration events that are organized every year since 1995, also served to help heal communities and renew social commitments to prevent the circumstances that lead to genocide, focusing particular attention on youth [12, 13]. In addition, a community justice system, known as Gacaca courts, was put in place from 2002 to 2012 to contribute to post-genocide healing as well as truth, justice and reconciliation [14–17].

Despite efforts to heal and reconcile victims and perpetrators, trauma remains prevalent in the country. Symptoms of Post-Traumatic Distress Disorder (PTSD), for example, have been reported by Rwandans who were children and adolescents during the genocide [18], and observed among a large number of genocide widows [19]. Similarly, symptoms of PTSD and mood disorders (anxiety and depression) have been reported by both survivors and perpetrators, with a higher prevalence among survivors [20]. Moreover, results from the 2018 Rwanda Mental Health Survey recently found that more than 30% of survivors have major depressive disorders and almost 30% have PTSD compared to 12% of depression and 3.6% of PTSD symptoms in the general population [21]. Other significant consequences of the genocide that aggravate this trauma include poverty resulting from the destruction and looting of properties and lack of social support due to the disruption of social ties and high levels of mistrust between genocide survivors and perpetrators [22]. Symptoms of trauma are also being observed among Rwandan youth who did not physically live through the traumatic events of the 1994 genocide against the Tutsis [23, 24]. It is therefore timely to advance understanding of how trauma resulting from the 1994 genocide may be transmitted to youth as well as its effects on reconciliation.

This study was largely informed by the previous study which found that the trauma experienced by Rwandan mothers who survived genocidal rape was often transmitted to their children, suggesting that mental health needs linger decades after genocide [1, 8]. Further, much has been documented about the consequences of the genocide among Rwandans who were alive at the time and aware of what was happening during it. In addition, several studies have recently reported symptoms of PTSD among survivors’ offspring [25]. Previous studies from other settings than Rwanda, suggest that PTSD among the next generation is associated with offspring’s direct exposure to traumatic events or to trauma that is transmitted to them intergenerationally [3, 25, 26]. In the context of Rwanda, some studies have concluded that intergenerational trauma among descendants of Tutsi survivor mothers is the result of trauma transmitted to them epigenetically [27]. What remains unclear is whether there are other possible pathways by which trauma is passed down to subsequent generations and the extent to which such dynamics may impact reconciliation processes. This article addresses this gap by exploring perceived mechanisms of trauma transmission to youth born to genocide survivors after the genocide and how these mechanisms might affect reconciliation processes in Rwanda.

### Trauma and its conceptualization in Rwandan context

Previous authors have defined trauma in different ways. In their work on victimhood, Fassin and Richtman referred to trauma as the collective imprint left by horrific past experiences that may have happened years or generations ago [28]. Historically, trauma is understood as the high prevalence of disease exhibited by people who have been exposed to threatening events such as mass trauma-colonialism, slavery, war, and genocide with possibilities of affecting multiple generations that come after its original occurrence [29]. As understood by the local population^2^, trauma or *Ihungabana*^3^ is also a milder form of distress as a result of a troubling past [30]. Furthermore, trauma can be referred to as *Ihahamuka* meaning trauma crisis, which is partly explained as the manifestation of PTSD or sometimes confounded with panic attacks accompanied by fear and shortness of breath [19]. In Rwanda, traumatic events from the genocide include mass killings, looting of properties, being hunted, being injured, witnessing the death of others and hiding in unsafe places such as bushes or in marshlands. Other events included sexual violence which was used as a weapon of war against women during the genocide [31–33]. In this article, *Ihungabana* or trauma and *Ihahamuka* or trauma crisis [34] will be used interchangeably.

### Intergenerational trauma

It has been argued that in countries that have faced extreme violence and/or genocide, trauma becomes part of people’s history, affecting survivors as well as their friends, family members, and communities, and is passed on to subsequent generations. Such transferred trauma is known as intergenerational trauma. Those who did not personally experience the originating traumatic events can experience intergenerational trauma as well. Rather people who experience intergenerational trauma “absorb” the unresolved trauma or psychological burden from those who directly experienced the traumatic events [24]. We herein define intergenerational trauma as the manifestations of a range of parental trauma in the lives of the generation that was born after the 1994 genocide in Rwanda.

Four major theoretical approaches to understand the transmission of trauma from parents to their children are: psychodynamic and relational, culture and socialization, family systems and communication and biological or epigenetics mechanisms [23, 24]. The psychodynamic or relational dimension emphasizes the transmission of trauma through unresolved or repressed parental emotions that are unconsciously and indirectly displaced onto children by parents. Consequently, exposed children are at risk of developing problems and behaving as if they went through the traumatic events themselves [24]. Parents and their children affected by this process develop strained relationships characterized by poor ties among them [23, 35].

The sociocultural and socialization approach argues that trauma is transmitted through parenting and modelling. This transmission can occur both consciously and directly through social learning. Such a learning involves human development processes over the life span, which are shaped by embodied interactions with others and within a person’s surroundings [36]. For family systems theory, it is suggested that trauma can be transmitted through family enmeshment and communication styles used by families of survivors. Such transmission can be attributed to a social pattern whereby survivor families often prefer to maintain close relationships with other survivor families. Thus, children grow up in an environment where the past is constantly made present [23]. Others have theorized that parents transmit trauma to their children through biological or physiological processes (e.g., electro-chemical processes in the brain). Consequently, children get exposed at risk of inheriting parental traumatic stress and are specifically vulnerable to mental health problems resulting from genetic aetiology due to changes to a parent’s biology from being exposed to traumatic events. Along these lines, research has shown that the negative effects of PTSD on parenting and epigenetic programming may in turn lead to the descendants of mothers with PTSD being at higher risk of suffering from PTSD, anxiety or depression [35, 37, 38].

### Understanding of reconciliation in the context of intergenerational trauma

Reconciliation is the development of trust, change or re-establishment of relationship between people, communities and societies with the goal of fostering partnership between conflicting parties based on reciprocity and mutual responsiveness [39]. Trust building is crucial for promoting reconciliation because safe relationships or co-existence between victims and their perpetrators is essential [40]. Furthermore, there is evidence from African contexts that rebuilding relationships between former enemies depends on: perpetrators and victims physically meeting; perpetrators seeking pardon and, ideally, be forgiven by the victims; perpetrators acknowledging their wrongdoings; and survivors reconciling with their past traumatic experiences [41, 42].

Although there is no model yet theorizing the interconnection between intergenerational trauma and reconciliation, studies on traumatic experiences among people in post-conflict societies have shown that trauma potentially affects reconciliation processes especially among adults who witnessed the traumatic events [18, 21, 43]. Trauma can deeply destroy interpersonal relationships especially when perpetrators fail to repent and show remorse of their traumatic past acts committed towards the victims. Remorse by perpetrators should include elements of acknowledging other’s pain, guilt and shame resulting from causing such pain, accountability and admission of complicity of the crimes committed, in order to facilitate the process of mourning and working through the past hence ensure forgiveness and reciprocal recognition of historical past between the two groups [44–47]. Failure to acknowledge other’s pain may cause feelings of injustice, humiliation, and diminished sense of individual and collective identity which may persist over generations [48]. Next to being consequences of human right violations among older generations, these feelings are eventually identified as potential factors that characterise intergenerational trauma [49].

Additionally, findings from a study conducted with youth in Croatia suggested that a sense of victimhood coupled with receiving threats from an out-group to one’s life or to the lives of significant others was associated with negative emotions towards the out-group and less propensity towards reconciliation. The study also showed that perceived constructive parental communication was associated with higher propensity towards reconciliation among youth belonging to the out-group. The same study suggested that young people who perceived their parents as using constructive and non-aggressive relational communication (versus verbal aggression) had a greater propensity towards reconciliation despite a high sense of victimhood [50]. This suggests that these dynamics may be further elucidated in the context of post-genocide Rwandan society.

## Methodology

### Study design

An exploratory qualitative study was conducted in two districts of the Eastern Province of Rwanda, Bugesera and Gatsibo, as well as Kigali city between July and November 2019. Topic guides for IDIs and FGDs were drafted, piloted, adjusted, and finalized to include the topics of intergenerational transmission mechanisms of trauma, the meaning of reconciliation and the relationship between trauma acquired by youth to the reconciliation process in post-genocide Rwanda. Each category of study respondents had its own specific topic guide.

### Respondents and study settings

Sixty-five respondents participated in the study. The respondents consisted of 36 genocide survivor parents, 19 post-genocide descendants of survivors, two district mental health nurses and eight mental health and peace-building professionals from Kigali.^4^ Although the study included survivor parents and descendants of genocide survivors, the descendants and parents were not matched dyads^5^.

All parents lived in Rwanda before, during and after the 1994 genocide. The respondents were recruited from five organizations: four local non-governmental organizations that worked closely with genocide survivors and one public institution. At the time of the interviews, almost a quarter of the survivor descendants interviewed still had both parents, while others were living with one parent, usually the mother. Most fathers had died due to the genocide or its consequences^6^.

### Data collection

Four trained and experienced assistant researchers with backgrounds in psychology, sociology, social work, and public health, collected data. Data collection techniques were semi-structured IDIs and FGDs. Each was 40–90 minutes in length. Most IDIs with descendants of survivors were held at their residence, and most interviews with professionals were conducted at their organizations’ offices while FGDs among parents were conducted at a safe location close to the respondents’ neighbourhood, based on their choosing. A brief demographic table outlining data collection techniques, numbers of study respondents in each category group, as well as demographic data such as age range and education level is provided below to give clarity of respondents involved in the study (see Table 1 Data collection summary). All data was collected in Kinyarwanda^7^ and later translated from Kinyarwanda into English while transcribing them verbatim for analytical purposes.

**Table 1:** Summary data collection techniques by category of respondent, numbers, age range and education level

Data collection technique	Category of study respondents	Number of respondents	Year of birth	Education level^a^
FGD	Genocide survivor parents	36	1942–1987	P4-S2
IDIs	Youth descendants of survivors	19	1995–2000	P4-S2
IDIs`	Mental health and peacebuilding professionals	10	1968–1983	U3-M4

^a^ P: Refers to Primary (level), S: Refers to secondary school or senior (levels), U: University (level), M: Master’s level.

### Data analysis

An inductive thematic analysis approach, guided by the research questions, drove the data analysis. This approach prioritizes the analysis of data without necessarily having a developed coding framework [51]. The approach helps to build knowledge on psychological, emotional, and social processes, such as the relationships between trauma and healing, based on people’s lived experiences, perceptions, and the impact of the narrative exploration of such phenomena [52].

IDI and FGD transcripts were independently read and re-read several times by each of the three authors for analysis and reporting of most recurrent patterns of meaning across the data set. Each IDI transcript was initially coded manually by the first author while the two other authors cross-checked emerging codes. When any different or new codes were detected, all authors discussed them until agreement on meaning was reached. Emerging themes and sub-themes were assigned to corresponding chunks of text across all the transcripts. Based on the themes and codes, the authors discussed observed patterns to generate general conclusions, which were recorded in writing, without necessarily developing any codebook. Final overarching themes appear below as the subheadings in the findings section.

### Ethics

All methods of the current study were applied in alignment with the regulations and standards of the Helsinki Declaration [53]. In compliance with national standards for human subject’s research ethics, an approval note (No. 1674/RBC/2018) was obtained from the Rwanda Biomedical Center (RBC) prior to the start of fieldwork. To protect the privacy of the respondents, pseudonyms were used after analysis. Respondents were also assured their names would be de-identified throughout reports and publications to protect their privacy. Study objectives were explained and informed consent was obtained from all subjects and this prior to taking part in IDIs or FGDs. In addition, it was explained to all respondents that the files containing the information they provided would be kept confidential.

## Findings

The findings of this study present experiences and perceptions that youth, parents, and/or professionals narratively shared in relation to the relevant themes, as well as exemplar quotes to illustrate key findings in detail. The first section showcases everyday living conditions of the descendants of survivors in their homes as shaped by the vulnerability of parents and various repercussions of parental trauma on the life of the young people. The second section describes perceptions on mechanisms through which genocide-associated trauma among parents is passed on to their young descendants. The third section shows the impact that trauma among youth may have on reconciliation — a crucial element in the process of ensuring future peaceful cohabitation between genocide survivors and perpetrators. The headings reflect the identified themes.

### Everyday life of survivor descendants

Genocide has had a myriad of consequences on the family with effects on the well-being of survivor families. Some youth grew up in uprooted families and have lost many extended family members. They expressed sadness about not having a family (place) to visit, especially during the holidays, as other children of their age typically do. Some children have parents who are physically disabled and/or traumatized by violent experiences during the genocide. Some parents developed chronic diseases and were unable to adequately respond to their children’ needs across the lifespan. The most prevalent illnesses among parents reported by youth were HIV/AIDS, *Ihahamuka* during the genocide commemoration period, and/or *Ihungabana* during other months of the year. The ill health of parents is perceived to cause youth to both feel deprived of parental affection due to both physical and emotional absence and growing up in impoverished families.

#### Growing up with distressed parents

Youth reported that their everyday life is marked by distressing health conditions that remind them that they would *not* have to face such suffering if the genocide had not taken place. According to young respondents, they carry the burden of living with traumatized parents as well as not living a life to its full potential. They repeated several times that they grow up in families that are emotionally and financially affected, hence leading them to act as their own mothers and sometimes their own fathers and/or taking care of their vulnerable parents by dropping out of school, working for money, and performing some of the parents’ household activities. Divine, shares how her role shifted:

*Consequences are many. I am not happy because of how I see her (*the mother*). If my mother is always sick, I cannot go anywhere. I cannot look for a job because I am the one to take care of her. I cannot go to school. Whenever I get money, I give it to my mother to pay medical bills. Other consequences are that I do my mother’s work, which is greater than what I can do under normal circumstances. ***Divine**, **# A young girl, IDI**.

Growing up in such conditions, which youth perceive as abnormal heavily, weigh on their everyday life and activities.

#### Changes in the family during the genocide commemoration period

Respondents across all categories also described a change of mood in the familial milieu during the genocide commemoration period. Observed changes were associated with a deep sorrow amongst parents and family members remembering the loss of their loved ones killed during the genocide. Isolation of parents, silence within the family and harsh behaviours towards children are the main characteristics cited. Despite physical parental presence at home, regular life routines are often disturbed, as parents tend to emotionally return to their past in ways that are a more or less visible to their descendants.

Moreover, interpersonal relationships, which might seem normal and positive between children and their parents, and between parents themselves, become irritated. One parent from a mixed marriage^8^ family gave an example of irritation in the relationship:

During the commemoration, he (her husband) always says: *you always go in your things, which are endless, instead of staying at home and carrying out some household chores. By the way, for how long you will keep walking after the past and dead people. This causes problem in relationship. Mathilde*, # parent, FGD.

Parent-child communication is seen as dominantly affected by warnings. Youth repeatedly highlighted that their parents warn them especially in terms of behaving carefully towards others and being home before it gets dark. Youth expressed that these warnings result from parents’ fear and worries about their children’s safety, assuming that they may be harmed by perpetrators of crimes against deceased family members who some survivors still mistrust.

*During the genocide commemoration period, there are many rules at home. My mother warns us that we should be at home early if we want to be safe. My mother is no longer the mother we know. I feel bad, but she looks worried too. I feel against her [mother] but I respect her, though I do not feel happy of that*. Kim, **#** A young boy, IDI.

Though the genocide commemoration period brings challenges at family level, on the one hand, young respondents perceive the commemoration events as beneficial and believe that it enables them to join their families in remembering their departed loved ones. They also learn about their familial genocide experiences that are normally silenced outside of the period and related events. On the other hand, youth in this study dislike hearing how their parents have been victimized. They expressed that they get abruptly exposed to such experiences that are sometimes vivid when parents are suffering from *Ihahamuka*, which increases significantly during this period. Parental behaviours and warnings during the commemoration that result from their suffering, often create barriers between young descendants of survivors and their parents, as well as between them and their peers from the perpetrator’s side. Sandra’s experience illuminates this:

*When they play commemoration songs or talk about genocide experiences, he* [the father] *is not happy. He doesn’t talk to us nor look happy as usual. He is not free to talk to us*. Sandra, # A young girl, IDI.

### Mechanisms of trauma transmission

Findings of this study have revealed four perceived possible mechanisms of trauma transmission. Those mechanisms include transmission of trauma through biological means, through silence and through disclosure of traumatic experiences and everyday contact with a traumatized parent.

#### Transmission through biological means

When asked about the trauma transmission mechanisms, parents and professionals narratively reported their perception that one of the transmission mechanisms can be the transmission of maternal trauma through blood during pregnancy and/or through breastmilk. For instance, one woman who was pregnant during the genocide explained that when perpetrators attacked their hiding area she became scared and the child in her womb stopped moving until her fear diminished. Parents also argued that a baby born from a traumatized mother may develop similar symptoms as his/her mother, namely fear, sorrow and pain as explained by some of the mothers:

*I think those symptoms [of trauma] are transmitted to the baby when still in her/his mother’s womb, maybe through maternal blood. Although the baby did not see the mother with those symptoms of Ihungabana, but after birth, you will gradually observe him/her developing some of the symptoms you presented when you were carrying him/her during pregnancy*. Cansilde, # parent, FGD.

However, this type of transmission was relatively unknown among young study respondents.

#### Transmission through everyday contact with (a) traumatized parent (s)

Respondents across all categories mentioned that trauma among descendants of survivors can also result from behaviours these descendants observe from own parent (s), during their everyday contact or due to how a child is taken care of or treated by the parent during breastfeeding, bathing, or feeding activities. One of the professionals explained it like this:

*There is a common saying that “history repeats itself” or “history may be transmitted from generation to generation’’ not because it is genetic, but due to environmental factors such as those in which traumatized parents raise their children. For instance, if the parents are traumatized, live with sorrow, always sad, or in pain as result of atrocities committed against them during the genocide, the child who grew up seeing such parents in that mood, will also live a hopeless life, sad and unsocial with the likelihood of culminating into severe trauma (Ihungabana rikomeye).* Ange, # A psychologist, IDI.

What is reflected in the account of the psychologist in the above quotation, was reiterated by one young respondent:

*Whenever she developed mental trauma and cried, I would also cry, until they would take me away from her; after that, some people would remain counselling me, while others would take her to the hospital. After a few days, I could see her brought back in a stable condition. That is when I would also feel relieved. Then, I would stop thinking about that. Josée, # A young girl, IDI*.

In addition, parents believed they may also transmit their *Ihungabana* to their children through physically abusing them. Children who were brought up by such parents were perceived as ending up developing similar maladaptive^9^ behaviours and resulting symptoms of *Ihungabana*. Some of the manifestations of trauma among children were seen to resemble their parents’ symptoms. One of the parents who attended a FGD gives examples of commonly observed symptoms of *Ihahamuka* among youth:

*To lose a sense of direction, keeping quiet, lack of sleep, losing appetite, feeling rude and talking to others harshly; others look mad, they may look as if they have sorrow, others complain about headaches that do not respond to analgesics/medications, while some others have deep thoughts about the past and withdrawal from others. Cancilde, #* A parent FGD Bugesera.

Although most of the symptoms of *Ihahamuka* among youth are like those of adults, respondents also identified a difference. For instance, children who manifest reviviscency of past experiences do not express names, especially those of attackers, whereas this is spontaneous among adult survivors. Moreover, parents and professionals perceived that *Ihungabana* and *Ihahamuka* are mostly apparent among youth whose parents are severely traumatized and those who silence their genocide experiences. Still, even though youth recognize symptoms of trauma among adults and peers, half of the youth in this study doubted that trauma could be transmitted intergenerationally to those who were not yet born at the time of the genocide.

#### Transmission of trauma through silence of (parental) genocide experiences

Narratives across all the data and from all categories of respondents showed that some survivor parents seemed reluctant or found it difficult to share their traumatic experiences with their own descendants despite the eagerness of their children to know about their parents’ past. Multiple factors contributing to silence among parents were reported. In some cases, parents silenced the truth about the information sought by children as one of their strategies to deal with or stop their children from inquiring so much. Other parents chose to silence the past through making up stories that would help their descendants understand the past without explicitly sharing their past experiences. In doing so, parents act on the belief that making up stories can help to mitigate the risks of full disclosure, namely feelings of revenge among youth.

Silence around past experiences was given meaning by youth as well. Youth expressed that some parents are unable to recount their genocide experiences due to emotional distress hence inability to verbally respond to their children’s quest for answers. More so, youth have noticed that some of their parents may simply respond to their children by saying that they do not know how they survived, especially when children inquire about specific parental past experiences. One of the parents talked about these types of silencing:

*One day I went with my child to visit a memorial site, but they would not allow him to enter because he was still underage. Then we moved around the building. I was showing him the bodies of people who were killed during the genocide. As we walked, he asked me, “What are those sticks?” I said those are bones of the limbs. “Then where are the muscles and skin like ours?” I did not have an answer for that; but when he kept asking me the same question, I told him that the perpetrators who killed them went away. I told him like that because I did not want him to ask whether they are among our neighbours, otherwise he would later cause trouble in our neighbourhood.* Geneviève, *#* A parent, FGD.

From the point of view of professionals, parents resist telling their genocide story to children because they have not yet been able to come to terms with their own traumatic past. They argue that traumatic experiences have impaired parents’ ability to give the traumatic past a meaning and find appropriate words to describe it. Professionals associated this challenge with the inability to integrate trauma as well. Avoidance of discussing genocide experiences with children helps parents to protect themselves from the emotional pain that comes with recounting such experiences. Yet, suffering among parents is perceived as the source of suffering among children. One of the professionals who also is a mother illustrates this dynamic:

*Because witnessing the genocide events has caused some parents to feel as if they already died, some parents avoid telling their children that they once died and were then reborn. This is because they think it is not necessary to empty themselves into their children*. *For me as well, I think there is a need of reserving some parts of genocide experiences because they may affect children’s lives.* Julia # A psychologist, IDI.

Albeit various reasons for silence among parents were given, the study findings suggest that silence is a potential mechanism through which trauma of the past is transmitted. For instance, many youths believed that when parents make-up stories, they may inadvertently introduce more emotional risk than they hope to mitigate. More so, some survivors’ descendants may experience vicarious trauma due to sympathizing with parents when they struggle to silence their past. Others may develop trauma through imagining what unknown genocide experiences might have been like. One of the young respondents explains how one can get *Ihahamuka* through the imagination when parents silence genocide-related inquiries:

*Even if parents do not like to tell us about the genocide, someone can imagine what his/her parents went through and make a link between that imagined experience and what he/she sees in the memorial sites, and immediately creates a scenario in his head leading up to trauma crisis. This may happen especially when one sees the parent remembering the bad experiences that marked the country and go back to that time, simply because you asked him/her to tell you.* Parfait, # A young boy, IDI.

This imagination sometimes leads descendants to feel as if they lived through the events themselves, thus developing trauma symptoms very similar to those common among adult survivors.

#### Transmission of trauma through disclosure of genocide-related stories, experiences, and testimonies

Although some respondents emphasized that Ihungabana is transmitted through silence, disclosure of the past, or the opposite of silence, was also recognized as a trauma transmission mechanism. One parent reported:

*Whatever children hear [about the genocide], they keep it in their mind, and as they keep absorbing all those stories, they develop Ihungabana.* Geneviève, # A parent FGD.

Most youth reported that they have been exposed to different sources of genocide related history. Such sources include, schools, through being exposed to genocide-related testimonies, mostly from individual survivors, at memorial sites during commemoration or remembrance events or through (over) hearing unexpectedly such information as part of conversations among parents, elder siblings, or other family members. Conversations about the genocide are myriad around and during the annual genocide commemoration period. However, they judge the content of stories related to genocide experiences that they acquire from such sources to be less complete.

Moreover, some stories provided are more traumatizing than others. The most traumatizing aspect of the past is learning about the inhumane actions of perpetrators against victims, such as those resulting in the death of a relative or the genocide experiences of one’s own parent(s). With sympathy towards his parents, and in an emotionally disturbed tone with tears in his eyes, Damien, a young man, shared the following:

*My mother usually tells me about it and even when we go to the memorial site, they tell us about some victims and we find that they are our relatives…Since I knew it, it was hard for me to accept the way they died. It is painful when a razor cuts you - imagine how hard it is to accept that they have cut someone’s head or leg with a machete*.

Even if some testimonies are more traumatizing than others, young respondents recognized that not all genocide testimonies can lead to *Ihungabana or Ihahamuka*. Most youth explained that it depends on how testimonies are shared, the psychological state of the people surrounding the child in everyday life, and the behaviours of the community members and the environment in which children grow up. For instance, some youth said that parents or family members express genocide experiences unwittingly when they are suffering from *Ihahamuka*. This is to say that they convey horrible stories through howls or screams, flashbacks and calling out to people unknown to the youth. Yet, youth consider these kinds of stories to be fragmented or incomplete. When the suffering person looks like they are in another world where they see things that other people do not and have difficulties interacting and communicating with the actual people around them, youth find it challenging to apprehend the full story being conveyed. And the content of such a story may itself be dangerous. Speaking plainly about potentially dangerous content is not the choice of *Ihungabana* victims, but rather the incidental result of overwhelming emotions following the remembrance of past violence committed against them. In this regard, psychologists perceived disclosure as harmful depending on the manner parents use to communicate traumatic experiences.

*The way parents narrate the story may end up psychologically traumatizing the children. For instance, telling the children how their father or uncles were being chased, how they escaped, how they found them where they were hiding, and how they cut them in pieces with a panga and so forth. That way of narrating stories of what happened during the genocide to children is the one that arouses emotions that may lead to Ihungabana among the youth*. Jeannine, # A psychologist, IDI.

The content of the story shared may also negatively affect their children. Accounts of parents revealed that when parents share their genocide-related stories in full, unrestrained emotional intensity with their children, trauma transmission is strongly enabled from parents to their young post-genocide descendants. Study findings suggest that this unlimited openness is commonly found among severely traumatized parents as well or among perpetrators who still display genocide ideology. Indeed, under such conditions, most youth are left grasping to understand what reasons could have led to such hatred and killings. Furthermore, disclosure by parents becomes more harmful when their traumatic experiences are repeated several times.

*…Another thing is that a parent can continue to implant those things in me by repeating what happened and who did it…Repeating its cruelty causes trauma for us.* Jacob, # A young boy, IDI.

What seems clear is that both the nature of content disclosed and the way it is disclosed by adults to younger people play a key role in intergenerational transmission of trauma from older to younger generation.

### Respondents’ way of understanding of reconciliation

Before asking respondents about the effects of trauma among the younger generation on reconciliation, they were asked about how they make sense of reconciliation. Respondents across all categories defined reconciliation as restoration of former relationships between genocide survivor and perpetrator families. This re-establishment of relationships requires seeking forgiveness from the side of perpetrators, which was valued and reported as an important and a key element in this process.

Moreover, respondents narrated that seeking pardon should be accompanied with reparations by perpetrators for what they damaged during the genocide. Reparation and seeking genuine forgiveness mean, for survivors and their post-genocide descendants, acceptance of moral responsibility, as opposed to the attribution of responsibility for genocide acts to the former government often espoused by perpetrators. Respondents defined moral responsibility as the acceptance of one’s own individual role/responsibility during the genocide and being accountable to ensure true repentance and genuine reconciliation between the victim and perpetrator.

Further related to reconciliation, finding the remains of their loved ones’ bodies for a decent burial was highly valued by both parents and young respondents. Burying the bodies of loved ones is seen as one of the remedies to heal the trauma of both parents and their young descendants. This is because a decent burial is also perceived as source of emotional relief and something that would help them to let the past go, facilitate them to forgive the offender, and make reconciliation more successful and sustainable. One respondent stressed how challenging reconciliation can be for traumatized people:

*After more than 20 years, nobody has ever come to tell me where my beloved relatives who were killed were thrown. No one told me: “You know I am the one who looted your home.” Yet, those are people we stay with in this village! Perpetrators have instilled a weevil of trauma in our souls! (Imungu y’umutima). If they happen to show me where my relatives are, that weevil of trauma is over from me, then I will forgive them.* Dancilla, *#* A parent FGD.

Reconciliation was not seen as possible when it involves one side —perpetrators only. Youth suggest that for reconciliation to be possible, survivors should also offer forgiveness to those who wronged them and apologized. This process of seeking and offering pardon was reported to be one of the durable strategies to enable former enemies to genuinely re-unite and truly live peacefully after the genocide.

### The effects of (intergenerational) trauma on reconciliation

While assessing the impact of intergenerational trauma among youth on reconciliation processes, parents and professional respondents reported that the trauma of the past is being transmitted from adults to their young descendants. Additionally, it was perceived that traumatized young people cannot be open to reconciliation because those who are traumatized are still tied to the past due to losing hope for the future. Traumatized victims were perceived as facing difficulties of looking ahead because trauma was understood as somehow having the capacity to damage the possibility of seeing a future. Similarly, respondents valued having peace of mind as a prerequisite for healthy interpersonal relationships between people and for future peace.

Trust was elucidated as important in the reconciliation process as well. The findings revealed that a trauma victim may mistrust and feel afraid of people who harmed him/her in the past because of continuing to seeing their former perpetrator (s) as enemies who can attack and wrong them at any time.

Such feelings are commonly found among parents and young respondents. They expressed that some youth develop grudges after being traumatized by the genocide experiences of their parents. Other youth grow up with fear towards genocide perpetrators, especially when they know them. Divine said:

*When I see someone who committed Genocide, like the ones I was shown, I am worried that he can kill me. When I see him, I run away because I am afraid of being killed.* Divine, # A young respondent, IDI.

From the point of view of some youth who do not know who their family perpetrator is, they are at risk of mistrusting any other community members beyond their family members. They suspect most of the adult people of their parents’ age to be genocide perpetrators. Such youth are the ones that live with fear that the genocide might happen again, at any time. Some of these youth appear isolated, unhappy, and mentally unstable and confused. Others are aggressive towards others, look depressed and limit interpersonal relationships with peers born of perpetrator parents. Professionals assert that someone who presents these trauma symptoms cannot take steps towards reconciliation. One young respondent has also confirmed this:

*No, it is not easy to talk about unity to such people who still have Ihungabana because they are still taken up by the past. It’s hard for that person to forgive. It requires a strong heart; so, they need to be counselled first to get relief from trauma, prior to telling them about unity and reconciliation*. Josée, # a young respondent, IDI.

The influence of parents on youths’ participation in reconciliation is seen as crucial. On the one hand, youth reported that reconciliation is possible among youth. On the other hand, they contended that the possibility of reconciliation is influenced by the willingness, degree of healing or of trauma and level of involvement of one’s parent(s). Young respondents, for instance, expressed that some parents are still disconnected from other community members and unable to forgive those who wronged them because of their trauma, and that this has an influence on youth as well.

*It is impossible for me to live peacefully with the killers’ families knowing that they are the reason why my mother is severely traumatized. When my mother gets trauma crisis (Ihahamuka), I get so sad. I start to focus on and isolate myself and get on my mother’s side*. *So, in this way*, *I think that parents may also influence us when it comes to reconciliation because they did not forgive and do not want us to forgive either.* Divine, # A young respondent, IDI.

Young people also expressed that such disconnected parents may possibly be attempting to protect their young descendants from harm by discouraging them from interacting with perpetrators as well as, perpetrators’ family members, and children. Similarly, reconciliation among youth was also thought to depend heavily on reconciliation among parents:

*Yes, it (Ihungabana) may have a negative effect on the unity and reconciliation process, and we have seen that any traumatized individual is not mentally well. Yet a mentally affected person cannot think of unity and reconciliation. Another issue related to that is that children take their parents as role models. So, if parents are not mentally stable, they will not think of unity and reconciliation. Likewise, if parents are not united, even their children will not think of unity and reconciliation with their fellow children or with other people*. Ange, # A Professional, IDI.

Despite the influence of parents on reconciliation processes —due to their unhealed trauma, hence inability to forgive—, the inability of some youth to participate in reconciliation processes appears to potentially be associated with intergenerational trauma. Additionally, this trauma was regarded to be intensified by the community environment in which these children grow up. For instance, the way perpetrators seek forgiveness was viewed as hampering the openness and commitment of descendants of survivors toward reconciliation. The perpetrators’ testimonies^10^ were judged by youth to be superficial especially when they (testimonies of perpetrators) downplayed their role and the severity of their wrongdoings by attributing their own involvement in genocide crimes to the former government. Descendants of survivors interpret such distancing from one’s crimes among genocide perpetrators as a form of denial and/or non-repentance which discourages them to forgive. Acknowledging their guilt in an unrepentant manner also create problems for survivor descendants:

*They [perpetrators] should say it in a humble manner. Sometimes they list what they did and people they killed, the target numbers of people they wanted to kill; and they do that proudly. So, that makes me sadder to think that they seek for forgiveness by telling stories but behave inappropriately. That causes a problem in me*. Jacob, # A young boy, IDI.

While some young respondents reported that their parental difficulties to forgive and reconcile create obstacles for them, they acknowledge that relationships between youth from both survivor and former enemy families seem better than that of their parents and that this is also the wish for some of their parents. Youth also endorsed this perspective reason and expressed that they are willing to have a good relationship with their peers from perpetrator families especially because the government has given them an example through treating all youth equally and without any discrimination^11^. However, youth stress that this willingness can only be possible under certain conditions. For instance, they pointed out that they couldn’t reconcile with the enemy before their parents do so because parents are the primary victims among whom reconciliation should first take place, to pave the way for descendants thereafter. Reconciling with the opponent’s side before a parent reconciles with their perpetrator is perceived by youth as a re-traumatizing act toward their parents.

Some respondents supported the idea that young people from both survivors’ and perpetrators’ families should be assisted and facilitated to join and attend platforms together to help them to potentially have good relationships today and in the future. In addition, respondents suggested that all post-genocide generations should have their space for healing from their trauma and develop a sense of connectedness to overcome trauma, hence becoming able to secure a peaceful future.

## Discussion

This study explored the mechanisms of intergenerational transmission of trauma among young Rwandans whose parents survived the 1994 genocide against the Tutsis and effects of this trauma on reconciliation in the Rwandan context. Our findings suggest that trauma of the 1994 genocide against the Tutsis extends its effects to post-genocide descendants of survivors. Our results are similar to prior studies [39] and they suggest that such descendants of survivors inherit trauma of their parents through various mechanisms and that most of their trauma symptoms are to some extent similar to those of genocide survivors in general.

The transmission of trauma appears to take place most often within the family environment and during the genocide commemoration events and associated rituals. These findings are in line with the literature indicating that beyond affecting the primary victims or individuals, past stressful events may also have long-term and deep intergenerational effects on those who did not go through these events [54, 55]. The trauma transmitted to young descendants also has implications for reconciliation between young descendants of survivors and the families and children of genocide perpetrators.

The first perceived mechanism of trauma transmission identified by this study is biological transmission, reflecting the perceived possibility of the transmission of traumatic effects epigenetically. Though parents reported this possibility, and effects of trauma on fetoplacental interactions during prenatal periods were found in other settings [56], we did not examine this type of transmission due to the limitations of the study methodology. Our analysis is only informed by the perceptions of genocide survivor parents, post-genocide youth born of survivor parents and mental and peacebuilding professionals. Rwandan survivor parents perceived that they had transmitted their trauma, anger and aggression to children if they were pregnant during the genocide or during the course of child-rearing. This finding has resonance with the cultural notion of valuing blood and milk as the foundation of life. In Rwandan cultural cosmology, a parent gives life to her child through blood before birth and through breastfeeding a parent may transmit bad or good behaviours and attitudes to their children. This milk-giving is not limited to literal breastfeeding, but extends to the parenting style as a determinant of who the child will be in the future. From our understanding, the perceptions of respondents also reflect how vital milk and blood are in the Rwandan life and context. In accordance with previous studies [57, 58], this is illustrated by respondents’ perceptions that trauma from one generation to another can be carried through the human substances of milk and blood, which has been also concluded by Taylor: “*the healthy body is seen as a system of fluids in constant flow, and so are society and the entire universe. Illness, as well as social and cosmological disorder, is interpreted as the result of blockage or excessive flow… Flowing substances which represent the vitality and fertility of life include milk and blood*” [59].

The second identified mechanism is the transmission of trauma via parent-child interactions. This transmission probably results from everyday exposure of children to parental emotional suffering and growing up in families where parent-child attachment have been weakened by trauma [60]. We argue that developing *Ihungabana* or *Ihahamuka* symptoms among this category of youth is also somehow related to the identification by children of their parents’ experiences as their own, as reported by preceding researchers [61].

Both silence and open, mostly repeated communications of traumatic experiences are also perceived as potential mechanisms of trauma transmission. Findings revealed that youth whose parents silence their traumatic past are likely traumatized because they are unable to grasp and digest the genocide stories that they encounter from various other sources such as neighbours and commemoration events or because they spontaneously encounter fragmented or contradicting stories narrated by parents or perpetrators which mostly cause them to imagine more than what has happened [62].

As observed by our young respondents, some of them presented *Ihungabana* and/or *Ihahamuka* symptoms. These results agree with earlier studies. Trauma found among Rwandan youth might have resulted from being exposed to demonizing stories of survivors shared at memorial sites as this was reported by Münyas in a study conducted among Cambodian youth [63, 64], though settings differ. Moreover, in contrast to the usefulness of open communication in the study by Braga and colleagues [65], in this study disclosure of past related stories was mainly shared by people other than parents thereby contributing to *Ihungabana or Ihahamuka* symptoms due to overwhelm. In other cases, trauma emerges among youth when some parents have opened up about their past too explicitly, especially when disclosure involved negative emotions such as anger.

A family environment significantly marked by silence and social and emotional withdrawal fosters intergenerational transmission of parental trauma, as opposed to a supportive family environment that encourages more adaptive coping patterns such as perseverance and self-esteem/confidence among children [66]. Other factors enabling the transmission process include but are not limited to growing up in an environment where parents are vulnerable due to trauma or where survivors live next to their offenders, the annual commemoration events/rituals, parenting style and communication of overwhelming stories to children most often by family members and neighbours.

Additionally, there is a strong relationship between intergenerational trauma transmitted within families and the impacts of reconciliation processes (such as issues that were not solved by Gacaca courts), annual recurring memorialization rituals/events and educational interventions. This occurs mainly because the younger generation has grown up within these settings and is currently involved in reconciliation processes that sometimes evoke genocide memories. In turn, genocide memories reactivate the parents’ trauma that affects reconciliation in various ways.

Findings suggest that the aspects of trauma that parents most often transmit to their young children are feelings of mistrust and hatred towards perpetrator families, fear of being killed, hopelessness and a negative worldview with negative attitudes towards others that can lead to revenge or a cycle of violence. *Ihahamuka* symptoms were also observed among all three respondent groups: parents, professionals and a few young respondents who had themselves suffered from *Ihahamuka*. These findings provide new and additional evidence to consider alongside the findings of preceding studies [67, 68] that reported *Ihahamuka* among respondents who were born before the genocide and were thus assumed to have been reliving what they already went through.

There are some young respondents who denied that trauma resulting from the genocide could afflict them because they were not alive during the genocide. However, this denial does not impede the possibility of trauma development among the generation born after a traumatic past. This rather confirms the results of other studies [69] that found that despite the denial of trauma as a psychological coping strategy, the psychological pressure related to this denial can travel from one person to another, express and manifest itself through the recipient’s body. This denial of trauma by youth can also probably result from marginalization of trauma victims following the cultural connotation of trauma with madness or being crazy in Rwanda, hence causing some youth to deny it in order to save their image (as positive) in their communities as was found among young Cambodians too [70]. However, this stigma may also hinder youth from seeking mental health services that would support them to overcome trauma transmitted to them intergenerationally.

With regard to the linkage between intergenerational trauma and reconciliation, on the one hand, young respondents in our study showed willingness to forgive and reconcile with their peers from the perpetrator group and their parents supported reconciliation among the younger generation. On the other hand, youth resisted the possibility of this reconciliation as a way to protect their parents from re-traumatization, insisting that parents must forgive first to pave the way for youth. In other words, the youth perspective is: “*I will forgive after my parents did so”*. This point of view is likely related to the inheritance of severe family trauma, hatred and low levels of intergroup trust and forgiveness which is probably resulting from greater discrimination (in previous episodes of violence such as from 1959 to 1973) that have led to multiple trauma among parents over the years. Furthermore, these challenges among youth might be from the result of influences by parental discourse in the familial milieu [71].

Along the same lines, respondents emphasized that reconciliation can be possible after victims of past genocidal trauma are healed. Our findings revealed that the healing of emotional wounds is a prerequisite to personal well-being, to healthy and effective communication, including within families, and to reconciliation with others. These findings correlate with previous studies [72] and support the larger hypothesis that trauma healing can lead to development and is ‘a condition for peace’ [69, 73]. We suggest that healthy communication, which may lead to reconciliation, cannot be easily attained among descendants of survivors before their parents integrate/heal from their past trauma.

Study findings suggest that unhealthy communication among parents might be attributed to being silenced by trauma while wishing to provide testimony to others regarding what happened. When they speak, some survivors communicate overtly through their bodies, gestures, and uncoordinated speech. Sometimes this communication is unpredictable and can involve talk about people others do not see, as reported above [74]. From our point of view, this communicative manner can be understood as non-conducive for youth and a challenge to their development of constructive communication styles that normally contribute to facilitating reconciliation between them and their peers from the perpetrator families.

Despite the difficulties that youth are currently facing in terms of reconciliation, interventions are available to support youth in overcoming their trauma and helping them to reconcile with others. For instance, evidence suggests that interventions carried out through community-based healing groups may contribute to the open communication about past traumatic events within the family environment [75, 76]. Such interventions may also lead to improved psychosocial well-being, stable family environment, the promotion of peaceful cohabitation in communities along with the possibilities of hastening the unity and reconciliation process among both descendants of survivors and perpetrators. Therefore, creating safe spaces in order to help both survivor and perpetrator parents as well as their descendants to discuss their past as a support to healing from trauma may nurture improved parent-child communication, thus promoting improved inter-group relationships between parents which can also extend to the descendants of both.

Reconciliation efforts would benefit greatly from mobilizing genocide perpetrators to acknowledge their moral responsibilities, repairing the damage they caused and seeking genuine forgiveness to facilitate survivors to heal from trauma. We argue that these three elements (acknowledgment of own wrongdoings, repairing what was damaged and seeking forgiveness from the victims) are an essential remedy that contribute to healing trauma of gross human violation among victims. It is indeed the lack of these elements that hinders reconciliation.  These are conditions that will support and enable survivors to consider offering pardon to their perpetrators and to have the feeling of benefiting from justice. Youth may not easily engage with reconciliation effects if their parents are not healed from trauma. Healing from trauma, experiencing that the justice was rendered, being sought and offering pardon are crucial elements to ensure peaceful cohabitation after the mass violence. Where possible, creating spaces that would enable perpetrators and survivors to meet, discuss about the benefits of reconciliation as well as to seek and offer forgiveness in the presence of their young children would also have a positive impact on the way the youth understand the genocide and regard and engage themselves in reconciliation processes. This is because the parental role matters in the interpersonal relationships of their young children. In this respect, witnessing reconciliation of parents by descendants may lead to breaking the cycle of intergenerational trauma and hatred and ensuring sustainable peace across generations in the future. To achieve this, intergenerational dialogues that promote truth talking about parental individual genocide experiences, should be created as platforms where adults who have integrated their trauma do mentorship of youth. This will minimize provision of fragmented and contradicting information in the historical trauma discourse. They should also involve testimonies from families that were able to reconcile. Such positive testimonies may inspire youth not only to become people who acknowledge their wrong doings, but also to be people who can easily forgive. In addition, those who provided them serve as role models for the younger generation. Actually, this may help youth to understand that bad actions can be transformed into good ones, hence pave the way for youth to look positively towards intergenerational reconciliation for the better future. Additional value may be added by defining a framework for trauma healing among the post-genocide generation in mental health policy. For instance, youth-friendly centres, rehabilitation centres and schools are better places where a comprehensive package of trauma healing and reconciliation promotion can be delivered. In this regard, these settings should be considered for peace-building purposes.

Moreover, creating jobs and mobilizing youth to work together in small income-generating activities may foster cohesion, facilitating those who are stigmatized by their communities^12^ to become more socially connected and reintegrated. If not, expecting reconciliation among traumatized people can be unrealistic. Furthermore, though disclosure of the past is also among the mechanisms of trauma, it was clear that this transmission depends on the content shared and the way it is shared. In this regard, we suggest that communication of (the parental) traumatic experiences to children and teachings of the genocide history should consider their age, the content to share, how (the manner) and when to share those past experiences. Healing from individual and collective trauma is of utmost important in this process because healthy minds are needed to create peaceful societies.

### Study limitations

The findings of our study are limited to the perceptions and experiences of a limited number of respondents. Therefore, due to the size of the sample, these findings cannot be generalized. In addition, the views of perpetrator families were not included in this study due to time constraint. Research should explore intergenerational trauma and reconciliation among young descendants of genocide perpetrators and their parents as well. Furthermore there is a need for conducting a comprehensive study on the communication styles that Rwandan parents use while communicating past experiences, understanding the disclosure process, and exploring the appropriate age for children to be told about such traumatic experiences, as well as the appropriate manner to communicate such a past. Furthermore, our study did not focus on the dyads such as father-son, mother-daughter or a parent with own child transmission or verify effects on gender, although male parents were part of the study. We suggest that future studies should explore this too.

## Conclusion

The traumatic events related to genocide have severely affected the mental well-being of survivor parents. Such trauma is currently being transmitted to their post-genocide descendants and negatively affects their psychological well-being with possibilities of limiting their involvement towards reconciliation. Openness and willingness of youth towards reconciliation depends on their level of integrating trauma from the past into their present lives as well as on their parents’ healing and their ways of engaging with the reconciliation. Youth may not engage into reconciliation due to fear of re-traumatizing parents or due to mistrust towards families of perpetrators following their failure to apologize.

To better foster intergenerational reconciliation, we suggest that seeking pardon should shift from an individual (perpetrator) request and become a family responsibility, process, and commitment. This is because in many cases children consider their parents as their role models. Similarly, vulnerabilities of parents affect their children in one way or another So, seeking pardon and witnessing reconciliation between families by the younger generation may contribute to healing trauma among survivor parents and allowing descendants of survivors to reconcile with perpetrator families, thus breaking the cycle of intergenerational trauma while increasing the chances of having a reconciled post-genocide generation. This may in turn ensure future peace between individuals in communities as well as in the country as a whole.

Mental health professionals and peace-building organizations are called on to continue reinforcing the integration of trauma management into their services to support survivors, perpetrators and their descendants in order to succeed in this journey of rebuilding peaceful societies after mass atrocities. Specific programmes for strengthening unity and reconciliation processes and those aimed at healing among parents are needed to warrant reconciliation and promote harmonious relationships and peaceful cohabitation among former enemies and the descendants of both. Increasing the scope of these programmes among youth is also needed to foster mutual understanding, trust, mutual tolerance and to prepare for a smooth transition from parents’ painful past towards building a new future free from violence.

## Data Availability

Data are available from the corresponding authors and may be shared upon substantial and rational request.

## Footnotes

1 Tutsis is one of the three ethnic groups in Rwanda: Tutsis, Hutu, and Twa.

2 Local population means people currently living in Rwanda.

3 Ihungabana is the literal translation of Trauma in Kinyarwanda, a local language.

4 As most of their organizations are situated in Kigali city, the mental health and peace-building professionals were recruited exclusively from there.

5 Not from the same family, not a parent with own child.

6 Examples of consequences include beatings during the genocide and HIV/AIDS infection from the rape of a spouse. In one case, his family’s perpetrators killed a father after the genocide.

7 Kinyarwanda is the local language.

8 A couple of a genocide survivor female with a non-genocide survivor (hutu) husband.

9 Culturally perceived as inappropriate.

10 Currently, testimonies of perpetrators appear in public when asking for pardon, which happens mostly during the genocide commemoration period.

11 Examples given include attending the same schools, learning the same history, and using the same services, such as health facilities.

12 Stigmatized due to suffering from Ihungabana or Ihahamuka.
